# Chitosan Nanoparticle/Simvastatin for Experimental Maxillary Bony Defect Healing: A Histological and Histomorphometrical Study

**DOI:** 10.3390/biomimetics8040363

**Published:** 2023-08-14

**Authors:** Muna Alaa Alsaeed, Nada M.H. Al-Ghaban

**Affiliations:** Department of Oral Diagnosis, College of Dentistry University of Baghdad, Baghdad 10071, Iraq; nada.mohammed@codental.uobaghdad.edu.iq

**Keywords:** chitosan, simvastatin, maxillary bone, bone regeneration

## Abstract

Biomaterials such as chitosan and simvastatin (Sim) have been introduced to accelerate the extensive and multicellular biological process of bone healing. The aim of this study was to evaluate the bone healing potential of chitosan and Sim, alone or combined. Forty-two male New Zealand rabbits were divided into three groups: chitosan nanoparticles (ChN), Sim and chitosan simvastatin nanoparticles (ChSimN). Two bony defects were created in the maxillary bone. The hole on the right side received one of the experimental materials, while the other side was assigned as the control and left to heal without any intervention. Bone specimens were collected at 2 and 4 weeks and then taken for histological and histomorphometrical analyses. The histological findings revealed that ChN possessed the highest number of osteoblasts and osteoclasts at weeks 2 and osteocytes after 4 weeks. There was a significant difference between the two healing periods regarding all bone parameters across all groups. ChN stood out as the only group that had a significant difference in the count of all bone cells between the two periods, thus having the best potential in promoting bone healing.

## 1. Introduction

Traditionally, autologous or artificial bone grafting techniques were commonly used to treat bone fractures and deformities. However, due to the risks associated with bone replacement surgery, such as morbidity and infection, these procedures are not always recommended. An additional technique for bone repair and regeneration is the use of synthetic scaffolds that encourage osteoblasts to differentiate and produce new bone tissue to replace the damaged regions [1,2]. In general, the speed at which a bone heals depends on the extent of bone injury and soft tissue damage [3]. The bony fracture heals via a complicated, dynamic and diverse process, which includes inflammation, the formation of a soft tissue callus and the growth and remodeling of the bony callus [4,5].

Chitosan is a polycationic biopolymer with special chemical characteristics: a positive charge, the presence of a reactive hydroxyl group and an amino group, conferring this substance with outstanding bio-adhesion, biocompatibility and biodegradability abilities. Therefore, it was introduced as an active agent for treating wounds, cancer, diabetes [6], and ulcers [7], and is also used in dental therapies [8]. In addition, it has been utilized as a vehicle in the delivery mechanism of certain drugs [9]. In vitro studies indicate that chitosan has osteogenic qualities because it encourages stem cells to differentiate into osteoblasts that produce bone and encourages the development of bone colonies [10]. In addition to that, in vivo studies have indicated the osteogenesis potential of this agent [11]. The molecular weight and degree of deacetylation of chitosan have a significant impact on its properties, particularly when converted to nanoparticles [12]. According to previous studies, the molecular weight and DA of chitosan directly affect the biodegradation process, as this process is slowed at higher molecular weights [13] and prolonged at a higher DA (between 84 and 90%). A lower DA (between 82 and 65%) results in a faster breakdown, whereas highly deacetylated chitosan (over 85%) has a low degradation index in an aqueous environment and will decompose after a few months. This characteristic affects some biological characteristics of chitosan, including its potential for healing, an increase in osteogenesis and the process by which lysozymes break down biological systems [14]. Many researchers have focused on the ionic gelation technique of chitosan for developing nanoparticles, which can effectively package biological macromolecules, deliver the targeted medication into the body and release the drug gradually under controlled conditions [15]. When combined or homogenized with tripolyphosphate (TPP), a multivalent anionic polymer, chitosan is able to instantly turn into a hydrogel by forming intra- and intermolecular cross-linkages mediated by electrostatic attraction between the positively charged amino groups of chitosan and the negatively charged phosphates of TPP [16,17]. Polymeric nanoparticles obtain and maintain the drug’s appropriate therapeutic concentration at the site of action, which is the main goal of all pharmacological therapies. It has the ability to regulate drug release over an extended period of time, raising the therapeutic index of the pharmacological activity of substances [18]. Chitosan nanoparticles (ChN)have been widely utilized for bioactive compounds due to their high physicochemical stability, capacity to increase bioavailability, nontoxicity and potential targeting [19].

Simvastatin (Sim) is among the drugs used for reducing cholesterol via the inhibitory mechanism of 3-hydroxy-3-methylglutaryl coenzyme A reductase, which prevents the conversion of this enzyme to mevalonate; as a result, the process of cholesterol synthesis is inhibited [20,21,22]. It is considered a statin that is suitable for triggering bone development according to in vitro and in vivo studies [23]. The novelty of the osteo-promotive qualities of Sim is attributed to Mundy et al., in 1999, who concluded that Sim could encourage bone repair in the calvarial defect model [24]. This is attributed to suppressing bone resorption through decreasing the expression of TRAP and cathepsin K, preventing the fusing of osteoclast precursors and reducing the number of active osteoclasts [25]. Additionally, it increases osteogenesis through BMP-2 expression upregulation in osteoblasts via the inhibition of inflammatory cytokine tumor necrosis factor-alpha (TNF-α), which acts on preventing osteogenesis [26]. Further, it reduces the production of proinflammatory chemicals, consequently reducing acute inflammatory responses induced by the implantation of biomaterials [27,28]. Sim should be continuously and precisely delivered using the best medication delivery technique to the damaged area. This capacity is influenced by the loading method and materials of the delivery system [29,30,31]. Limited data are available about the combined effects of chitosan/Sim on bone healing. Thus, this study aimed to evaluate the efficacy of chitosan and Sim, alone or in combination, on bone healing.

## 2. Materials and Methods

### 2.1. Study Design and Setting

A total of 42 mature male New Zealand rabbits, aged 6 to 8 months and with an average wight of 1.5 to 2 kg, were used in this study. This study complied with the ethical standards of the College of Dentistry, University of Baghdad (protocol reference number 767). The animals were housed in private animal housing, where the health of all animals was checked and controlled. On the right and left buccal sides of the upper diastema, two intrabony holes, measuring about 3 mm in diameter and 4 mm in depth, were drilled into each rabbit. A split mouth design was made in which the right side defect was filled with the experimental material, while the left side defect was allowed to heal naturally. The rabbits were randomly divided into three groups, with 14 rabbits in each group: ChN group, Sim group and simvastatin chitosan nanoparticle group (ChSimN), taking into consideration that the left side for each rabbit in all groups was assigned as the control (Co). The rabbits were sacrificed at 2 and 4 weeks, with 21 rabbits for each period. During the whole experimental work, only one rabbit was lost and replaced with another one meeting the same criteria.

### 2.2. Interventions

The ionic gelation method was followed in the manufacturing and preparing of ChN, as previously described [32]. Briefly, low-molecular-weight chitosan (degree of deacetylation ≥ 90%, Glentham Life Science, Corsham, UK) was dissolved in a 0.01% (*v*/*v*) acetic acid (Thomas Baker, India) solution and stirred for 24 h to produce a 2.5 mg/mL chitosan solution. The pH was brought up to 5.5 using a 0.5 M sodium hydroxide solution, and the final concentrations were diluted in deionized water. The final concentration of tripolyphosphate (TPP) (DIDACTIC, Barcelona, Spain) in deionized water was 0.25 mg/mL. The TPP and chitosan solutions were filtered using a millipore membrane with a 0.45 um thickness. The TPP solution was then added dropwise (0.3 mL/min) to the chitosan solution, with vigorous magnetic stirring at room temperature. After that, the suspension was allowed to become a gel for 30 min.

The ionic gelation method using TPP as a crosslinking agent was also followed in the preparation of ChSimN [33]. A 9 mL chitosan solution in 1% *v*/*v* acetic acid (pH 3.5) was mixed with (5 mg/ 0.5 mL) of a saturated Sim ethanolic solution (Sigma-Aldrich Chemical Co., St. Louis, MO, USA). Then, 1 mL of the TPP solution was applied drop by drop, homogenizing the mixture for 8 min at 12,000 rpm and was further stirred for 2 h at 600 rpm. The created nanoparticles were separated via centrifugation at 18,000 rpm for 20 min.

To create a gelatin that was used for loading Sim, 5 g of gelatin powder was dissolved in 100 mL of water (Sigma-Aldrich, St. Louis, MO, USA) at 60–70 °C. The solution was applied with a micropipette and allowed to cover the whole tray. The granules were then chilled to dry out [34].

### 2.3. Surgical Procedure

**Figure 1:**
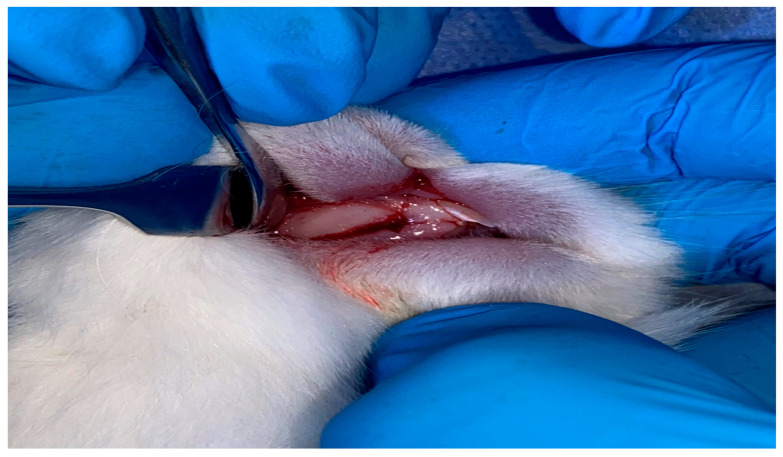
Bone exposure of the site of work.

Following the sterilization of all surgical instruments and towels, a general anesthetic solution containing 50 mg of ketamine HCl (20 mg/kg BW) and 20 mg of xylazine (2%) (0.2 mL/kg BW) was injected intramuscularly. Each rabbit received an injection of 20% oxytetracycline (1 mL/kg body weight) into the thigh muscle one hour before surgery, and the same dose was continued for one week (once daily) after surgery to prevent any infection. Additionally, to prevent the cornea from becoming too dry, a tetracycline eye ointment was used. A two-sided flap was created by making two surgical incisions in the natural edentulous gap (diastema of the rabbit), which is seen between the centrals and the premolars (Figure 1). The diastema distance was measured using a vernier caliper and the bony hole was made precisely in the midline. A bur stopper was placed on the surgical bur after measuring the required depth using a vernier caliper [35], followed by intermittent drilling with a small, round bur (size #010) and later a fissure bur (size #010), with vigorous irrigation. For all materials (ChN, Sim which is loaded on the gelatin, and ChSimN), 0.1 mg was applied using a spoon excavator into the defect, followed by approximating and suturing the ends of the flap using a black silk suture (DEMOPHORIUS; Limassol, Cyprus). Sacrificing animals was conducted using an anesthetic solution overdose. In order to collect the samples using a surgical disc and microengine, the premaxilla of each rabbit was dissected, and the specimens were prepared by removing all soft tissue and cutting the specimen in half (right experimental and left control) at the midline, with a minimum 5 mm distance away from the surgical site, followed by fixation in 10% fresh formalin for two days. After that, the samples were decalcified using a formic acid sodium citrate solution (125 mL of 90% formic acid, 125 mL of distilled water, 50 mg sodium citrate and 250 mL of distilled water) [36]. The decalcification solution was changed every 3 to 4 days, and the samples were routinely probed with a fine needle for inspection. Decalcification took place when the needle was inserted. The specimens were washed in running water for 30 min to remove any leftover acid [37]. Dehydrated by immersing in ascending concentrations of alcohol and clearing was carried out by immersing into two different changes of xylene for 15–20 min. Embedding was carried out by immersing each sample in molten wax, and after a few hours, the samples were moved to additional two or three wax containers in order to replace the xylene with paraffin. Then, the specimen was placed into the center of a metal block and wax was poured over it to produce sample blocks. Sectioning was carried out using a microtome, and 4 µm (approximately) sections about were produced in a buccopalatal cutting manner. Slide staining with H&E was performed by first passing of all sections through alcohol concentrations, then soaking in hematoxylin for 15 min, followed by washing in tap water for 1 to 5 min. They were then moved into 1% acid alcohol for 5 to 10 s, returned to tap water for further differentiation of the blue stain, and emersed in the Eosin stain for 1 to 2 min. Finally, they were rinsed in tap water, dehydrated using 100% alcohol and then cleared using xylene. Lastly, a slide cover was attached using DPX.

### 2.4. Outcomes

**Figure 2:**
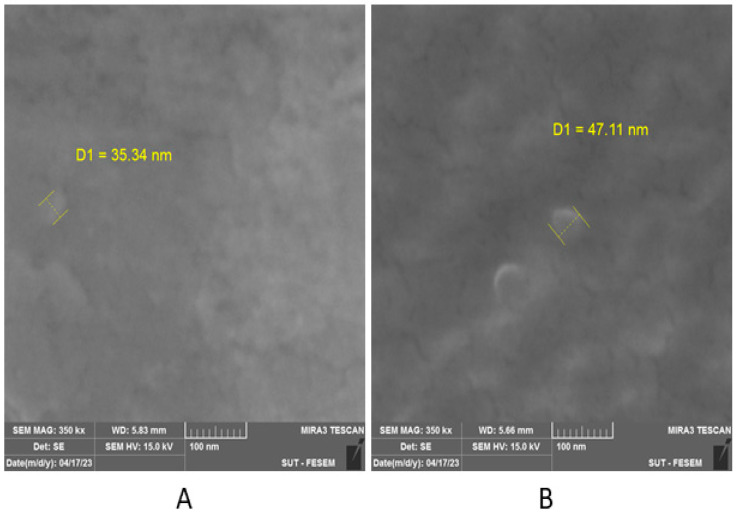
Particle size under field emission scanning electron microscopy. (**A**) The size of the nanoparticles of ChN, and (**B**) ChSimN.

**Figure 3:**
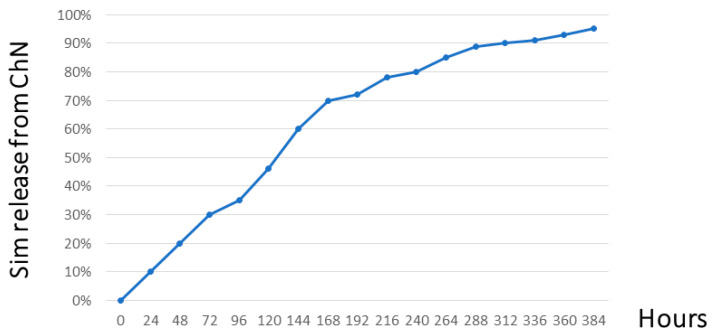
Drug release profile of simvastatin encapsulated by chitosan nanoparticles.

The particle size and shape for both chemically prepared materials (ChN and ChSimN) were measured employing a field emission scanning electron microscopy (Fe-sem) technique. The images for ChN showed irregular spherical-shaped particles, with a size of about 35.3 nm (Figure 2A), while the ChSimN appeared as regular spherical-shaped particles, with a particle size about 47.1 nm (Figure 2B). The zeta-potential for both materials was also measured. ChN had a zeta potential ranging from 25.8 mV to 31.1 mV, and the ChSimN particles had a zeta potential of about 24.6 mV to 28.5 mV which indicated that the particles of both materials were stable. The drug release potential for simvastatin entrapped by chitosan nanoparticles was measured using phosphate-buffered saline (pH 7.4), and found that it was 95.20%. After monitoring for 16 days, it reached the peak and then remained at the same level. This indicates that Sim was included and encapsulated within the NPs, and released in a sustained manner, which ascertains the quality of the manufacturing (Figure 3).

At two distinct healing intervals (2 and 4 weeks), osteoblasts (OBs), osteocytes (OCs) and osteoclasts (OCLs) were counted in histological sections stained with H&E and taken at ×40 magnification in four fields, and the mean was calculated. The bone marrow area (BMA), trabecular area (TA) and trabecular number (TN) were quantified using ImageJ, a computer program developed by the National Institutes of Health [38]. The distance in pixel was converted into a linear measurement unit (µm) before starting to measure the percent of (TA) and (BMA). The histomorphometrical analyzing was performed for the trabecular compartment only and it was ascertained as in the two weeks the cortical bone had not been formed yet, while at 4 weeks the cortical been had been distinguished and excluded from measurements by noticing the existence of few newly formed haversian systems. The region of interest appeared histologically under microscope as C shape highly demarcated from the old bone by reversal line. Histological examination was performed following the blinded examination technique to avoid any bias.

### 2.5. Statistical Analysis

The data were processed using the Statistical Package for Social Sciences (SPSS) version 26. The mean, standard deviation (SD) and range represented the descriptive data analysis. The distribution of data was confirmed via the Shapiro-Wilks test. The inferential data analyses included an independent *t*-test and analysis of variance (ANOVA) test. For multigroup comparisons, the least significant difference (LSD) was employed, considering significant difference when *p* < 0.05.

## 3. Results

A total of 42 rabbits were initially included in the study, with the loss of only one rabbit, which was replaced.

### 3.1. Histological Findings

#### 3.1.1. Two Weeks of Healing

**Figure 4:**
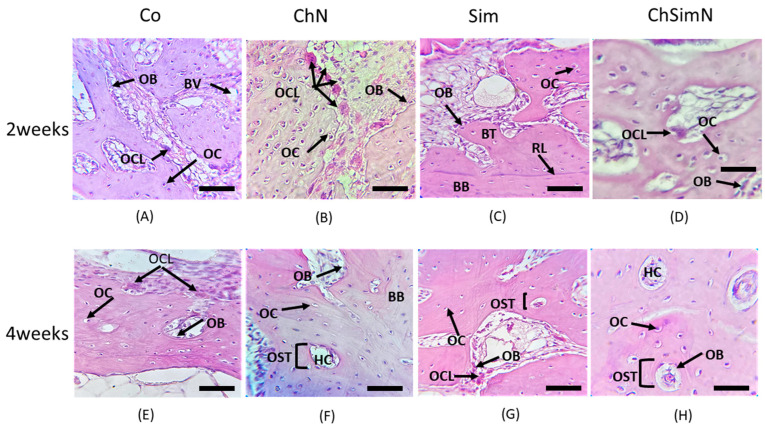
Histological section of the bone defect. (**A**) Control group (Co) at two weeks, showing osteoblasts (OBs), blood vessels (BVs), osteoclasts (OCLs) and osteocytes (OCs). (**B**) Chitosan nanoparticle group (ChN) at two weeks, showing multiple OCLs, OBs and OCs. (**C**) Simvastatin group(Sim) at two weeks, showing basal bone (BB) separated from the new bone trabeculae (BT) by a reversal line (RL), OCs and OBs. (**D**) Combination of chitosan nanoparticles and simvastatin(ChSimN) at two weeks, showing OCLs, OBs on the border of the new trabeculae and OCs embedded in the new bone. (**E**) Co at four weeks, showing mature bone trabeculae containing OCs, OBs on the border and OCLs. (**F**) ChN group at four weeks, showing OCs arranged in a circular pattern around the haversian canal (HC) forming the osteon (OST), OBs and RL separating the BB from the new bone. (**G**) Sim group at four weeks, showing OST, OBs and OCs. (**H**) ChSimN group at four weeks, showing OST, HC, OBs and OCs. H&E × 40. Scale bar = 50 µm, *n* = 42.

**Figure 5:**
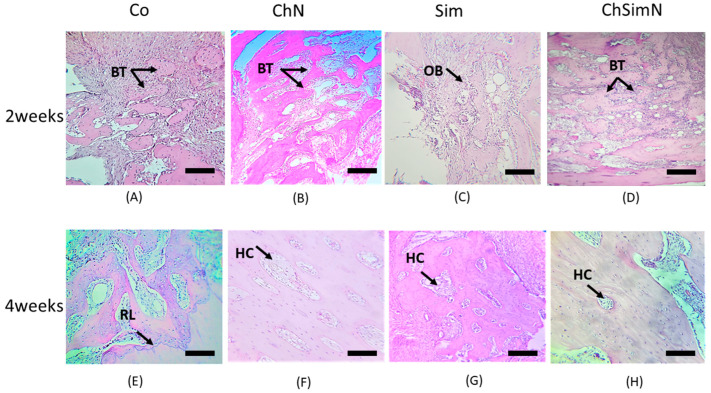
Histological section of the bone defect. (**A**) Control group (Co) at two weeks. The black arrow indicates new bone trabeculae (BT). (**B**) Chitosan nanoparticle group(ChN) at two weeks, showing newBT. (**C**) Simvastatin group(Sim) at two weeks. The arrows indicate osteoblasts (OBs). (**D**) Combination of chitosan nanoparticles and simvastatin (ChSimN) at two weeks. The arrow shows new BT. (**E**) Co at four weeks, showing mature BT separated from the basal bone by a reversal line (RL), which is indicated by the arrow. (**F**) ChN at four weeks, showing mature bone. The arrow indicates a haversian canal (HC). (**G**) Sim group at four weeks, showing HC. (**H**) ChSimN at four weeks, showing HC. H&E × 10. Scale bar = 100 µm, *n* = 42.

The histological picture of the ChN group at the defect site compared to the other groups revealed a high number of newly formed bone trabeculae encompassed by OCs, along with functional OBs on the border and OCLs residing the lacunae overlying the bone surface (Figure 4B and Figure 5B). On the other hand, the ChSimN group had a high number of newly formed trabeculae compared to the Sim and Co groups, but less than the ChN groups. OBs and OCLs were also evident, but at lower numbers than in the ChN group (Figure 4D and Figure 5D). Sim showed an osteoinductive property through the presence of osteoid tissue surrounded by osteoblasts near the material remanent. New bone trabeculae were also present in this group, with few osteoblasts and osteocytes (Figure 4C and Figure 5C).

#### 3.1.2. Four Weeks of Healing

The histological findings of the ChN group showed thicker trabecular bone compared to the other groups, containing a high number of small, regularly spaced OCs arranged around the haversian canal, OBs that line the border of the trabeculae and few OCLs appearing in certain places with a noticeable decrease in the bone marrow area (Figure 4F and Figure 5F). The ChSimN group showed that the bone marrow sites were also reduced in size, surrounded by a mature thick trabecular bone compared to Co and Sim. Entrapped within these trabeculae, small OCs had a circular pattern around the haversian canal in this group and OBs lined the inside of the trabeculae (Figure 4H and Figure 5H). Sim also showed a thick trabecular bone, but to a lesser extent than ChN and ChSimN (Figure 4G and Figure 5G).

### 3.2. Histomorphometrical Analysis of Bone Architecture

**Figure 6:**
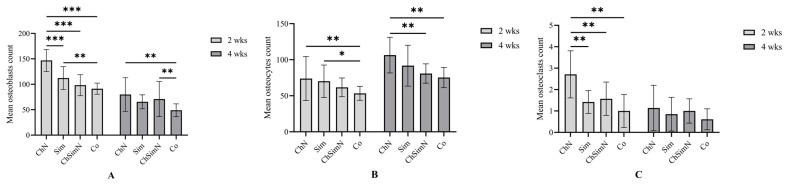
A bar chart representing the mean and standard deviation of bone parameters after 2 and 4 weeks, *n* = 42, unit of measurement (um). Counts of (**A**) osteoblasts, (**B**) osteocytes and (**C**) osteoclasts after 2 and 4 weeks. * *p* < 0.02, ** *p* < 0.002, *** *p* < 0.001.

After 2 weeks, ChN showed a significantly higher number of OBs than the other groups. In addition, the Sim group exhibited a significantly higher OB number than Co. After four weeks, the number of OBs in Co was significantly lower than the ChN and ChSimN groups (Figure 6A). Upon analyzing the mean of OCs at 2 weeks, Co showed a significantly lower number of cells compared to ChN and Sim. After four weeks, ChN had a significantly higher number of OCs compared to Co and ChSimN (Figure 6B). After 2 weeks, the result revealed that ChN had a significantly higher number of OCLs compared to Sim, SimChN and Co (Figure 6C).

**Figure 7:**
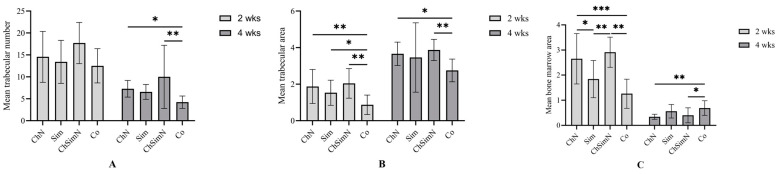
A bar chart representing the mean and standard deviation of bone parameters after 2 and 4 weeks, *n* = 42, unit of measurement (um). (**A**) Trabecular number, (**B**) trabecular and (**C**) bone marrow area at both intervals. * *p* < 0.02, ** *p* < 0.002, *** *p* < 0.001.

Upon analyses of the mean TN, the result suggested significant differences between the groups at 4 weeks and nonsignificant differences at 2 weeks. At week 4, the TN of both ChN and ChSimN was significantly higher than that of the Co group (Figure 7A). After 2 weeks, all experimental interventions exhibited a significantly higher mean TA than the Co group. At week 4, the results revealed that the TA of ChN and ChSimN was significantly higher than that of the Co group (Figure 7B). At week 2, the results showed that the BMA of both Sim and Co had a significantly lower mean value than that of ChN and ChSimN, while at week 4, Co showed a significantly higher mean for the BMA than ChN and ChSimN (Figure 7C).

Following intergroup comparisons, it was concluded that Ch had a significantly higher number of OBs and OCLs after 2 weeks than 4 weeks, while the mean of OC number was significantly higher at week 4. Sim had significantly more OBs after 2 weeks than 4 weeks, while Co exhibited a significantly higher OB number at 2 weeks and OCs at 4 weeks. The mean of OCs was significantly higher after 2 weeks for ChSimN. All the bone healing parameters (TN, TA, BMA) showed significant differences between the time points across all groups (Table 1).

## 4. Discussion

A bone defect refers to the loss of integrity of the bony structure, mainly attributed to trauma, bone tumors, degenerative illnesses, infections, osteomyelitis and a number of congenital disorders. Normally, relatively small bony defects are self-limiting and heal spontaneously. However, when the defects/lesions are extensive, healing is challenging for restoring bone homeostasis. Therefore, application of different interventions aims toward accelerating the healing process. Emergence of nano-drug delivery systems represented a promising approach to treat bony defects due to their unique physical, chemical and biological features [39]. Chitosan and its nanoparticles, in particular, are an attractive choice as a delivery system for controlled release due to exceptional biocompatibility, biodegradability, adsorption characteristics, stability, low toxicity and easy preparation [40]. The beneficial effects of Sim, especially on osteoinduction and osteogenesis, have been vastly reported [41,42]; therefore, it was included as one of the interventions, along with chitosan, in this preclinical trial. Rabbits were chosen for this study due to their ease of handling, brief lifespan and cost-effectiveness. Generally, rabbits are one of the most popular first-hand options for preclinical investigations [43,44]. In addition, they possess advantages over mice or rats in terms of evolutionary similarity to humans in blood volume, responsiveness and other resemblance. Further, they are considered superior laboratory animals, since they may closely mimic human physiological traits in biomedical research [45]. The edentulous space between the incisors and premolars was selected to be the experimental site because during mastication, this region is most likely to experience mechanical stress. This enables the model to accurately reflect the in vivo healing of intraoral jaw bone abnormalities [46].

Histological and histomorphometrical analyses revealed that all three experimental groups exhibited a higher rate of bone formation than the Co group in a time-dependent manner. Exposure of bony defects to ChN resulted in significant differences in the trabecular BMA and TN after 2 weeks compared to the controls. This was consistent with previous results reported by Jafarzadeh et al. [47]. Interestingly, the number of OBs and OCLs was significantly higher in the ChN group compared to ChSimN. This indicated the reducing OB differentiation capacity of ChSimN [48], suggesting an antagonist effect of Sim when combined with ChN. This was further demonstrated by the significantly higher number of OCs that were derived from OBs in association with ChN than ChSimN, which is consistent with results obtained by Ghadri et al. [49]. This notion is further supported by the anti-osteoclastogenesis effect of Sim, which could be responsible for reducing OCL populations. These findings disagree with those of Gallinari et al. [48], which could be explained by the differences in the delivery system, inclusion of calcium hydroxide and utilizing an OB cell line derived from osteosarcoma. The results of this study also indicate the better bone formation capacity of ChN compared to ChSimN. This fact was previously reported by Chen and coauthors (2018), who showed an improved bone formation capacity as a well-known feature of cross-linked chitosan scaffolds [50]. In fact, combining ChN and Sim not only compromised the biological effects of ChN, but also failed to improve any features of Sim alone. However, this disagrees with Cruz et al. [51], which may be attributed to the difference in the carrier additive material. These results were also inconsistent with Xue et al., who used a different study model, with a dissimilar site and higher concentration of Sim [52]. For instance, combining Sim with melatonin and the human allograft showed an improved bone healing capacity after 4 weeks [53].

Other findings showed that defects treated with ChN exhibited significantly higher BMA and bone-remodeling cells compared to Sim. This highlighted the efficiency of ChN for accelerating bone maturation, as previously suggested by Almashhadi and Alghaban [54].

Regarding the Sim group, at week 2, the results showed better effects for bone formation compared to the Co group, which agrees with the results of Zhao et al. [55]. However, after 4 weeks, the results of the study showed that there was no effect of Sim when compared to the Co group, disagreeing with the results of Karanikola et al. [56]. The variance in the results is mostly attributed to the difference in the study model and experimental site. These findings also contravene with the conclusions of Papadimitriou et al. [57]. This difference may be attributed to the use of different specific carriers, i.e., bovine bone graft and hydroxyapatite combined with calcium sulfate, and the extra-orally located bone defect.

After 4 weeks, the results also showed a higher rate of bone formation in ChSimN compared to the Co group in terms of TN, TA and BMA, and this is supported by the results of Delan et al. [33].

All groups showed a normal pattern of bone healing at different rates. This process includes the conversion of OBs that into OCs after 4 weeks, as they became entrapped into their matrix. Additionally, as much of the woven bone had already been transformed into lamellar bone, the number of OCLs decreased at week 4, and the newly formed bony trabeculae fused together, reflecting the rise in the TA and diminution in the BMA.

The established mechanism of Sim to increase bone formation is related to its role in stimulating BMP-2 expression. This in turn increases osteogenesis and decreases the process of osteoclastogenesis, thereby preventing the fusion of OCL precursor cells by lowering tartrate-resistant acid phosphatase gene expression; as a result, the process of bone resorption is inhibited [21,58,59,60]. It also increases vascular endothelial growth factor expression, vascular endothelial cell proliferation and differentiation, all which promote angiogenesis [29,61] and lower inflammatory reactions by reducing the production of proinflammatory substances, including interleukin 6. Consequently, this reduces acute inflammatory reactions brought on by the implantation of biomaterials [27,28]. As a drug delivery system, ChN is the most suitable form of chitosan derivative for prospects in the field of drug delivery [40]. In this study, nanoparticles were created by physical cross-linking using TPP. This method was used specifically because no heat was needed, soft mixing was used and no organic solvents were added [62]. The use of nanoparticles allow for controlling medication release, lowering dosages which reduces adverse effects, and improving drug stability, thereby boosting effectiveness and bioavailability [40].

The limitations of this study include the short healing period. Extending the assessment interval may give more definitive results considering the difference between the action of the groups. In addition, the use of immunohistochemical staining for certain markers, such as the transforming growth factor β or BMP, was not conducted and is recommended for future studies. The interaction between ChN and Sim should be further investigated to elucidate the antagonistic effect observed in this study. However, this work demonstrated the effect of ChN and Sim alone or in a combination in the oral cavity, which mimicked a real patient scenario. This is important, as a surgical procedure within the oral cavity is challenged by possibility of contamination and occlusal load. Application of bone-inducing materials in dental surgical procedures, such as the placement of dental implants, would significantly improve the outcomes. However, the current results should be interpretated with caution and confirmed by further studies to address any limitations before being generalized.

## 5. Conclusions

The use of ChN was associated with the best bone healing outcomes, while combining ChN and Sim failed to produce a similar effect. This combination could either reduced the bone healing capacity of ChN, Sim or both, requiring further studies to elucidate the mechanism behind this finding.

## Figures and Tables

**Table 1:** Intergroup comparison differences at both durations.

		Co Mean ± SD	ChN Mean ± SD	Sim Mean ± SD	ChSimN Mean ± SD
Osteoblasts	2 weeks	91.33 ± 10.9	146.85 ± 21.6	112.32 ± 22.5	98.28 ± 20.7
	4 weeks	48.92 ± 12.6	79.92 ± 33.2	65.51 ± 13.7	70.85 ± 34.5
	*p* value *	0.001	0.001	0.001	0.102
Osteocytes	2 weeks	53.35 ± 9.7	73.78 ± 30.5	70.14 ± 22.6	61.71 ± 13.0
	4 weeks	75.31 ± 14.0	106.42 ± 24.7	91.75 ± 28.3	80.85 ± 13.4
	*p* value *	0.001	0.048	0.14	0.019
Osteoclasts	2 weeks	1.0 ± 0.77	2.71 ± 1.1	1.42 ± 0.53	1.57 ± 0.78
	4 weeks	0.61 ± 0.49	1.14 ± 1.06	0.85 ± 0.89	1.0 ± 0.57
	*p* value *	0.066	0.02	0.18	0.15
Trabecular number	2 weeks	12.52 ± 3.9	14.57 ± 5.8	13.42 ± 4.9	17.71 ± 4.7
	4 weeks	4.23 ± 1.4	7.28 ± 1.9	6.57 ± 1.7	10.0 ± 7.2
	*p* value *	0.001	0.008	0.004	0.038
Trabecular area	2 weeks	0.87 ± 0.53	1.87 ± 0.93	1.53 ± 0.69	2.04 ± 0.81
	4 weeks	2.75 ± 0.62	3.66 ± 0.64	3.46 ± 1.9	3.87 ± 0.58
	*p* value *	0.001	0.002	0.038	0.001
Bone marrow area	2 weeks	1.26 ± 0.58	2.65 ± 1.0	1.84 ± 0.74	2.91 ± 0.6
	4 weeks	0.69 ± 0.29	0.34 ± 0.1	0.56 ± 0.27	0.4 ± 0.37
	*p* value *	0.001	0.001	0.003	0.001

Co: control, ChN: chitosan nanoparticles, ChSimN: chitosan simvastatin nanoparticles, Sim: simvasatin. * Significant difference at *p* < 0.05 using independent *t*-test.

## Data Availability

Data are available from the author upon reasonable request.
